# Studies of antimicrobial resistance in rare mycobacteria from a nosocomial environment

**DOI:** 10.1186/s12866-019-1428-4

**Published:** 2019-03-19

**Authors:** Sónia Gonçalves Pereira, Susana Alarico, Igor Tiago, Diogo Reis, Daniela Nunes-Costa, Olga Cardoso, Ana Maranha, Nuno Empadinhas

**Affiliations:** 1 Center for Neuroscience and Cell Biology (CNC), University of Coimbra, 3004-504 Coimbra, Portugal; 2 Institute for Interdisciplinary Research (IIIUC), University of Coimbra, 3030-789 Coimbra, Portugal; 3 Centre for Functional Ecology (CFE), Department of Life Sciences, University of Coimbra, Coimbra, Portugal; 4 PhD Program in Experimental Biology and Biomedicine (PDBEB), Institute for Interdisciplinary Research (IIIUC), University of Coimbra, 3030-789 Coimbra, Portugal; 5 Faculty of Pharmacy, University of Coimbra, Coimbra, Portugal; 6 Chemical Process Engineering and Forest Products Center (CIEPQPF), University of Coimbra, Coimbra, Portugal

**Keywords:** Nontuberculous mycobacteria (NTM), *Mycobacterium mucogenicum*, *Mycobacterium obuense*, *Mycobacterium paragordonae*, *Corynebacterineae*, Antimicrobial resistance

## Abstract

**Background:**

Nontuberculous mycobacteria (NTM) are ubiquitous in nature and recognized agents of opportunistic infection, which is often aggravated by their intrinsic resistance to antimicrobials, poorly defined therapeutic strategies and by the lack of new drugs. However, evaluation of their prevalence in anthropogenic environments and the associated antimicrobial resistance profiles have been neglected. In this work, we sought to determine minimal inhibitory concentrations of 25 antimicrobials against 5 NTM isolates recovered from a tertiary-care hospital surfaces. Antimicrobial susceptibilities of 5 other *Corynebacterineae* isolated from the same hospital were also determined for their potential clinical relevance.

**Results:**

Our phylogenetic study with each of the NTM isolates confirm they belong to *Mycobacterium obuense, Mycobacterium mucogenicum* and *Mycobacterium paragordonae* species, the latter initially misidentified as strains of *M. gordonae*, a species frequently isolated from patients with NTM disease in Portugal. In contrast to other strains, the *M. obuense* and *M. mucogenicum* examined here were resistant to several of the CLSI-recommended drugs, suggestive of multidrug-resistant profiles. Surprisingly, *M. obuense* was susceptible to vancomycin. Their genomes were sequenced allowing detection of gene *erm* (erythromycin resistance methylase) in *M. obuense*, explaining its resistance to clarithromycin. Remarkably, and unlike other strains of the genus, the *Corynebacterium* isolates were highly resistant to penicillin, ciprofloxacin and linezolid.

**Conclusions:**

This study highlights the importance of implementing effective measures to screen, accurately identify and control viable NTM and closely related bacteria in hospital settings. Our report on the occurrence of rare NTM species with antibiotic susceptibility profiles that are distinct from those of the corresponding Type strains, along with unexpected resistance mechanisms detected seem to suggest that resistance may be more common than previously thought and also a potential threat to frail and otherwise vulnerable inpatients.

**Electronic supplementary material:**

The online version of this article (10.1186/s12866-019-1428-4) contains supplementary material, which is available to authorized users.

## Background

Hospitals are major sources of infectious agents with 7 to 10% of all inpatients estimated to develop at least one hospital associated infection (HAI) during their admission [1–3]. In addition to the debilitated health conditions rendering patients more susceptible to infections, hospital environments represent added risks inflicted by antibiotic resistant opportunistic pathogens [3, 4]. The World Health Organization (WHO) recently issued its first ever list of antibiotic-resistant ‘priority pathogens’, the most prevalent and antibiotic resistant bacterial pathogens associated with nosocomial infections [5]. WHO emphasized “Mycobacteria was not subjected to review for inclusion in this prioritization exercise as it is already a globally established priority for which innovative new treatments are urgently needed” [5]. Most *Mycobacterium* species are environmental saprophytes designated nontuberculous mycobacteria (NTM) to be distinguished from those that cause tuberculosis [6]. Their ability to infect humans and to colonize man-made environments including hospitals with inadequate disinfection of water distribution systems, as well as an overall resistance to antibiotics and high prevalence of risk factors in the population namely chronic diseases and advanced age, all merge into a potential and serious health threat [7]. The growing number of NTM infections in the lung, soft tissue or skin of susceptible individuals, combined with their known association with nosocomial infections and outbreaks, has brought up a general awareness towards this bacterial group [7]. While clinical laboratories routinely screen for the presence of some important pathogens others are often neglected. These include fastidious and slowly growing pathogens or opportunists. For example, the clinical significance of NTM isolation besides the most prominent pathogens of the group (such as MAC or *M. abscessus*) is not totally understood, especially in lung disease. Most NTM are non pathogenic for healthy people, but almost all can be responsible for opportunistic infections in susceptible individuals. In cases of isolation of NTM usually considering normal colonizers or contaminants, patients require careful clinical evaluation, taking into account factors such as the patient’s immunologic status and the site of infection to determine the significance of the isolate [8, 9]. Potential pathogens can colonize hospital settings but the awareness of their presence is low because they are not routinely screened. In fact, there exists a bias in screening since only about 2% of microorganisms grow well in standard clinical culture media and are considered significant [10]. Some of the undetected pathogens emerge as cause of atypical infections frequently associated with relevant drug resistance, highlighting the necessity for urgent measures for their routine detection and control. For example, contamination of ICU inanimate surfaces and equipment has been identified as a contributing source of transmission of pathogens to ICU patients in outbreaks [11].

Members of the genus *Mycobacterium*, which in December 2018 comprised 198 valid species [12], are among a restricted gallery of the most resilient organisms we know of as they can withstand multiple stress conditions such as high temperature, oxidative stress, nutrient deprivation and prolonged desiccation [6]. Other related bacteria from the suborder *Corynebacterineae*, namely *Corynebacterium* spp., *Nocardia* spp., *Rhodococcus* spp. and *Gordonia* spp. can also be the cause of atypical infections in susceptible individuals but their routine identification has been neglected in the clinical practice as well [13, 14].

The aim of this study was to address the extent of antimicrobial resistance in strains of NTM and other *Corynebacterineae* isolated from a nosocomial environment. Although health authorities neglect the fact that the prevalence of NTM infection is seriously underestimated in the European Union in general [15] and in Portugal in particular [16], only a strong commitment to NTM research will allow proportional responses to this health threat.

## Results

### Distribution of NTM and other actinobacterial isolates, identification and phylogenetic studies

Samples were collected from different sites of 4 hospital wards in 3 sampling events as previously described [17]. All isolates were recovered after 2 or 4 weeks of incubation in Middlebrook 7H10-PANTA medium and none after 6 weeks incubation. Of the actinobacterial isolates 10 belonged to the suborder *Corynebacterineae,* their correct phylogenetic identification and antibiotic susceptibility pattern were the focus of the present study, to address the extent of antimicrobial resistance in *Corynebacterineae* isolated from a nosocomial environment. The other 24 isolates belonging to genera *Dermacoccus*, *Kocuria*, *Microbacterium* and *Micrococcus* (all non-*Corynebacterineae*) were not identified to the species level and their antibiotic susceptibilities were not examined in this study.

**Fig. 1:**
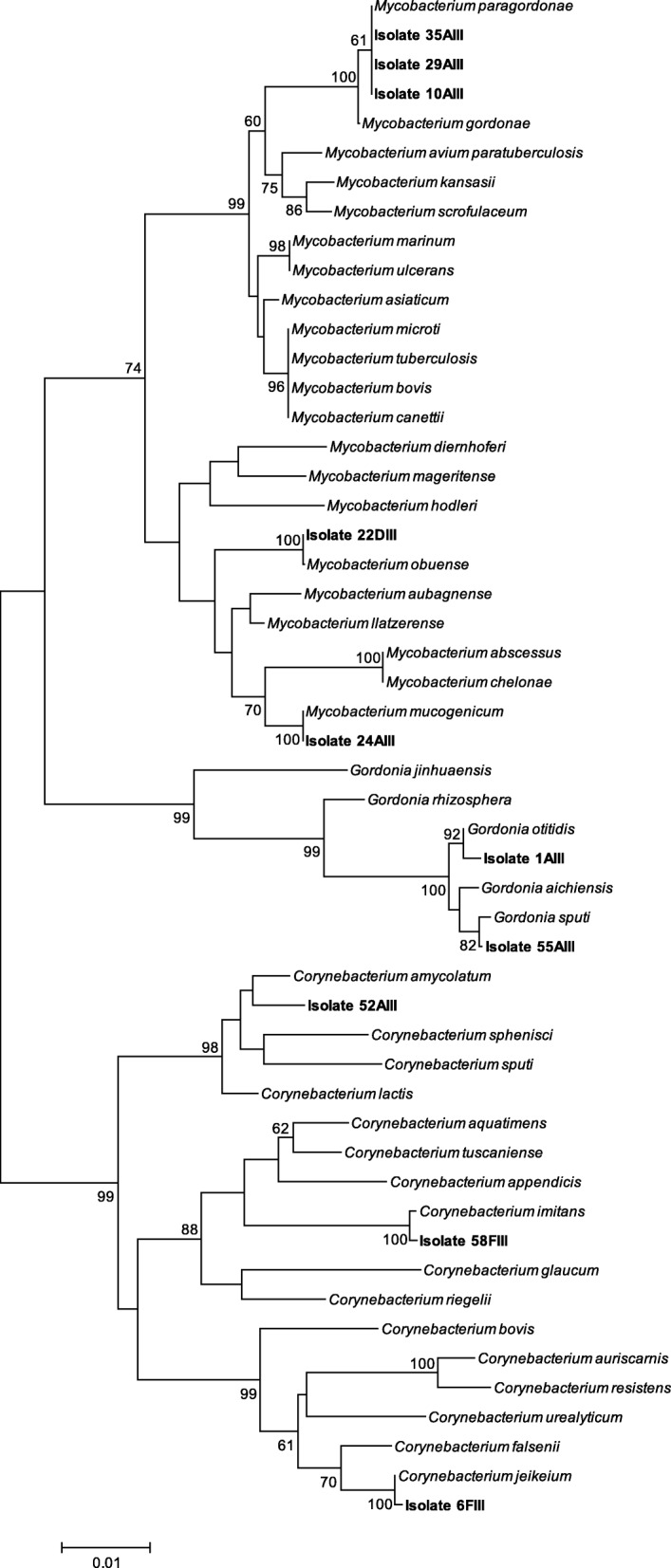
Phylogenetic tree based on a comparison of the 16S rRNA gene sequences of isolates and their closest phylogenetic relatives belonging to the *Corynebacterineae* genera *Mycobacterium*, *Corynebacterium* and *Gordonia* (16S rRNA gene sequences of type strains available from GeneBank) (also see Additional file 1: Figure S1, Additional file 2: Figure S2 and Additional file 3: Figure S3). The tree was created using the neighbor-joining method. Bootstrap values above 60%, for 500 replicates, are given at branch points. Bar, 1 inferred nucleotide substitutions per 100 nt

As inferred from 16S rRNA phylogenetic analyses, 3 isolates of the 10 *Corynebacterineae* were related to species *Corynebacterium jeikeium*, *C. amycolatum* and *C. imitans* and other 2 isolates were closely related to species *Gordonia otitidis* and *G. sputi* (Table 1, Fig. 1).

**Table 1:** NTM and other *Corynebacterineae* members isolated from different hospital sites (adapted from [16]). Phylogenetic trees in the present study confirm that isolates 10AIII, 29AIII and 35AIII are probably *M. paragordonae*

Isolate	Closely related species	Ward	Amenity
1AIII	*Gordonia otitidis*	Hematology	Restroom light switch
6FIII	*Corynebacterium jeikeium*	Hematology	Bed table
10AIII	*Mycobacterium paragordonae*	Hematology	Therapy room bench
22DIII	*Mycobacterium obuense*	Urology	Restroom sink
24AIII	*Mycobacterium mucogenicum*	Urology	Restroom light switch
29AIII	*Mycobacterium paragordonae*	Renal Transplant Unit	Therapy room bench
35AIII	*Mycobacterium paragordonae*	Renal Transplant Unit	Bed
52AIII	*Corynebacterium amycolatum*	Medicine A	Bed table
55AIII	*Gordonia sputi*	Medicine A	Bed hand support
58FIII	*Corynebacterium imitans*	Medicine A	Bed table light switch

**Fig. 2:**
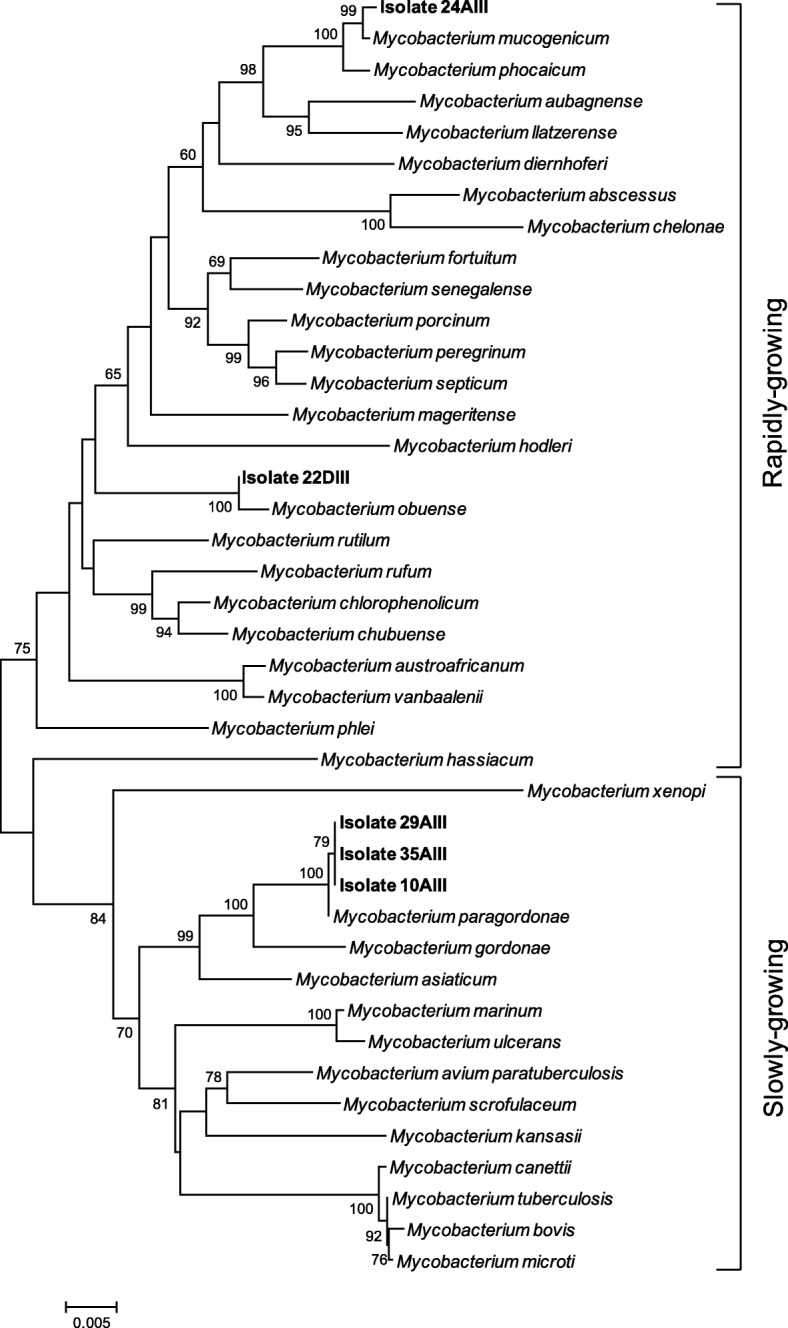
Phylogenetic tree computed from the concatenated nucleotide sequences of 16S rRNA, *hsp65* and *rpoB* from *Mycobacterium* isolates and from strains selected from databases, using the neighbor-joining algorithm. The tree includes 41 strains after checking the congruence from each single-gene tree (see Additional file 1: Figure S1, Additional file 4: Figure S4 and Additional file 5: Figure S5). The evolutionary distances were calculated by the Jukes and Cantor method [61]. Bootstrap values above 60%, for 500 replicates, are given at branch points. Bar, 5 inferred nucleotide substitutions per 1000 nt

Five of the *Corynebacterineae* were classified as members of the genus *Mycobacterium*, as determined from a phylogenetic tree of concatenated 16S rRNA, *hsp65* and *rpoB* genes (Fig. 2). One isolate was closely related to the species *M. mucogenicum*, other to *M. obuense* and 3 isolates were closely affiliated to the slowly growing species *M. paragordonae*, all nontuberculous mycobacteria (NTM) (Table 1, Fig. 2). Since *M. avium* is a commonly isolated NTM but was not detected in this study, control growth experiments to assess possible inhibition of growth by Middlebrook 7H10-PANTA were performed with some clinical *M. avium* isolates available in our collection, which ruled out such possibility (results not shown).* Corynebacterium* and *Gordonia* isolates were recovered from the Hematology and Medicine A wards (Table 1). NTM isolates were recovered from Hematology (*M. paragordonae*, 10AIII), Urology (*M. obuense*, 22DIII and *M. mucogenicum*, 24AIII), and Renal Transplant Unit (*M. paragordonae*, 29AIII and 35AIII) wards as previously described [17]. All but the *M. obuense* isolate were collected from dry surfaces/equipment.

### Determination of minimal inhibitory concentrations (MIC) of antimicrobials

MIC values of the 25 antimicrobials tested are indicated in Table 2. Considering the Clinical & Laboratory Standards Institute (CLSI) susceptibility interpretation for the antimicrobials included in the standards, the *M. obuense* isolate exhibited higher resistance levels and was the single isolate resistant to clarithromycin, which could be explained by the presence of the gene *erm* (erythromycin resistance methylase, accession number MG770427) in the draft genome sequenced in this study. On the other hand, *tet(V)* genes associated to tetracycline resistance were detected in the *M. obuense* and *M. mucogenicum* isolates (accession numbers MG770425 and MG770428, respectively) but only the latter was resistant to this drug (Table 2). *Mycobacterium paragordonae* isolates (*n* = 3) were susceptible to amikacin, ciprofloxacin, clarithromycin and linezolid, four of the antimicrobials recommended by CLSI to test drug susceptibility of slowly growing mycobacteria [18]. MICs for the other antimicrobials tested were not possible to interpret. All *Corynebacterium* isolates were resistant to ciprofloxacin, linezolid, penicillin and to imipenem, although the latter is recommended for *Corynebacterium* susceptibility testing despite the fact that no interpretative criteria are available (Table 2). Both *Gordonia* isolates were resistant to imipenem while only one of them (55AIII) was resistant to ciprofloxacin. These *Corynebacterineae* isolates were used in the susceptibility study to broaden the information about drug resistance in this particular phylogenetic group, as the information available is still extremely limited. High MIC values were observed for three of the PANTA antimicrobials tested against *Corynebacterium* and *Gordonia* isolates. On the other hand, azlocillin and polymyxin B showed the lowest MICs (Table 2).

**Table 2:**
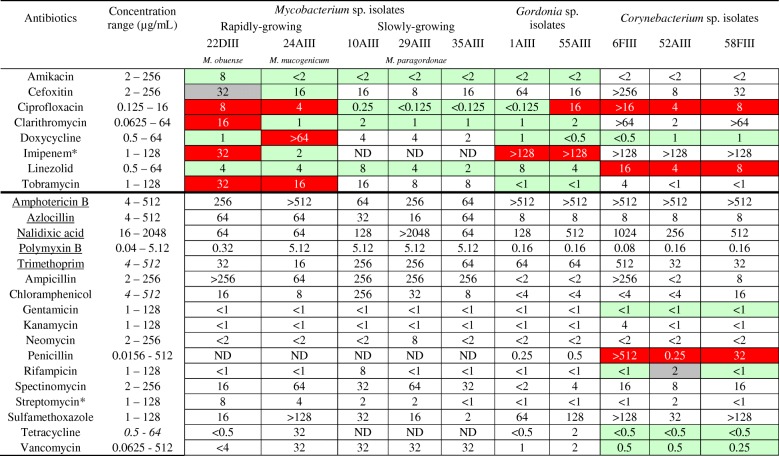
MIC values [μg/mL (U/mL for polymyxin B)] of 25 antimicrobials used for susceptibility testing of NTM and other *Corynebacterineae*

Resistant profile (red shading); intermediate profile (grey shading); susceptible profile (green shading), according to interpretative criteria [18, 68]. When no interpretive criteria are available only MIC values are reported. The dark line separates the antibiotics recommended by the CLSI to be tested for rapidly growing NTM (above the line) from the other 17 antimicrobials tested (below the line)

*ND* not determined

^a^Streptomycin is recommended for SGM susceptibility testing but no interpretative criteria are available. Imipenem is recommended for *Corynebacterium* susceptibility testing but no interpretative criteria are available. The 5 antibiotics with underlined names were utilized in the Middlebrook 7H10-PANTA supplemented isolation medium

All tested aminoglycosides inhibited the in vitro growth of NTM and of the other *Corynebacterineae*, even at their lowest tested concentrations. Tobramycin, one of the two aminoglycosides considered in CLSI standards for NTM susceptibility testing [18] was an exception, with the rapidly growing *M. obuense* and *M. mucogenicum* isolates displaying resistant profiles (Table 2). High MIC values were obtained for spectinomycin, with all isolates being able to grow in the range of 4 to 64 μg/mL with the exception of one *Gordonia* isolate (1AIII) (MIC < 2 μg/mL).

Although amphotericin B is an antifungal agent and no growth inhibition was expected for the bacterial isolates under study, the fact is that four of the NTM isolates were inhibited at the two highest amphotericin B concentrations tested (Table 2). Divergent results between NTM and other *Corynebacterineae* were also observed regarding chloramphenicol and azlocillin with all NTM appearing to be resistant to these antibiotics, while MIC values for *Corynebacterium* and *Gordonia* isolates were in general much lower. *Corynebacterium* isolates were susceptible to vancomycin and *Gordonia* isolates presented similarly low MIC values. As expected with vancomycin MIC values for the NTM isolates were high, except for *M. obuense* that was surprisingly susceptible to this antibiotic and also to polymyxin B (Table 2). All of the NTM and *Gordonia* isolates were susceptible to linezolid. *Corynebacterium* isolates 6FIII and 58FIII were extremely resistant to penicillin (> 512 and 32 μg/mL) (Table 2).

Results for ciprofloxacin, the only fluoroquinolone tested, ranged from high susceptibility in the 3 slowly growing NTM to the resistant phenotypes of the 2 rapidly growing NTM isolates (Table 2). Interestingly, high MIC values for ciprofloxacin were also obtained for the *Corynebacterium* isolates while the 2 *Gordonia* isolates had opposing results, one showed the lowest (< 0.125 μg/mL) and the other had the highest (16 μg/mL) MIC values measured.

## Discussion

Nosocomial infections are a major concern worldwide and represent an increase in hospital stay and treatment costs, particularly if associated with drug resistant pathogens [1]. The literature refers that probable dissemination vehicles between the pathogen niche (water faucets, medical instruments, fomites) and patients are mainly the healthcare providers [19].

Different wards of one hospital were sampled [17] and one third of the Actinobacterial isolates found belonged to the suborder *Corynebacterineae*, namely *Corynebacterium amycolatum* and *C. jeikeium*, *Gordonia otitidis* and *G. sputi* or *Mycobacterium mucogenicum*, all of which include potentially pathogenic strains previously implicated in human infections [20–22]. Of note was the fact that all samples were collected from sites with frequent human contact, placing these opportunistic pathogens easily accessible to patients, visitors and to healthcare providers. The surfaces of hospital amenities are considered important sources of pathogenic agents transmission [23] and the prevailing consensus indicates NTM opportunistic infections have environmental origin, although human-to-human transmission of *M. abscessus* is a factor of dissemination in cystic fibrosis patients [24, 25]. Isolation of *M. mucogenicum* has been mainly associated with hospital water distribution systems [26–31]. However, the isolate belonging to this species was recovered from a dry surface as were all the NTM strains studied here except the *M. obuense* isolate. Although *M. avium* strains are frequently isolated from waters, plumbing and showerheads biofilms, they are not, to the best of our knowledge, recovered frequently from surfaces such as those sampled in this study also possibly because their tolerance to desiccation appears to be low [32, 33].

The *Mycobacterium* strains recovered appeared to be relatively rare [17]. However, because their identification was initially based on 16S rRNA sequences alone, the putative *M. gordonae* isolates were now confirmed to be more closely related to the species *M. paragordonae* after concatenation of partial sequences of genes 16S rRNA, *rpoB* and *hsp65*, which provided a stronger species association confirmed by the construction of the corresponding phylogenetic tree. The *M. paragordonae* species was originally described in 2014 based on a clinical isolate from a patient with a pulmonary infection [34]. Since then *M. paragordonae* was only isolated twice, both from healthcare settings [35, 36]. The NTM species more frequently isolated from patients in Portugal in the last years were those in the *M. avium* complex, *M. gordonae* and *M. kansasii* [37]. Interestingly, our phylogenetic study shows that the three presumptive isolates initially identified as *M. gordonae* based on 16S rRNA sequencing [17], are in fact members of the recently described and rarely isolated species *M. paragordonae*, which raises questions about the true identity of clinical *M. gordonae* isolates, one of the species often recovered from patients in Portugal [37].

*Mycobacterium obuense* on the other hand appears to be common in soils and plants [38–40], but has been only rarely isolated from clinical samples [41–44] and, to our knowledge, there has been only one report of its isolation from a hospital environment [35]. Still, both species have been isolated from sputum of patients with pulmonary infections but, as is often the case for rarely isolated NTM [45], their clinical relevance remains uncertain. In addition to its clinical relevance and ability to cause a range of infections, *M. mucogenicum* has also been commonly implicated in nosocomial outbreaks [21, 46]. Indeed, its presence in the healthcare environment, if persistent, may pose a risk for patients. Although more prevalent than *M. paragordonae* and *M. obuense*, *M. mucogenicum* is still a rarely isolated species [15, 44].

The *M. mucogenicum* isolate showed a multidrug resistance (MDR) profile, at least to 4 different classes of antimicrobials (fluoroquinolones, tetracyclines, aminoglycosides and sulfonamides). This differs from what has been reported for the type strain *M. mucogenicum* DSM44625, which was found to be susceptible to ciprofloxacin, doxycycline and sulfamethoxazole [47], unlike the isolate in this study which was resistant to these 3 antibiotics. Furthermore, van Ingen and colleagues tested 15 *M. mucogenicum* clinical strains against a panel of 11 antibiotics and found the majority to be susceptible to rifabutin, amikacin, ciprofloxacin and clarithromycin [48]. The *M. obuense* isolate, also exhibited a MDR profile namely to ciprofloxacin, clarithromycin, imipenem and to tobramycin, unlike the *M. obuense* type strain ATCC27023 [47] and unlike a clinical isolate [48] both susceptible to ciprofloxacin, cefoxitin, tobramycin and clarithromycin. In our study, the *M. obuense* isolate was the only NTM resistant to clarithromycin. We have sequenced the genomes of the 5 NTM recovered (unpublished results), and *M. obuense* was the only to possess a classical *erm* gene [49]. To our knowledge, no *M. paragordonae* strains have been tested for antibiotic susceptibility prior to this work and the isolates tested here were susceptible to 4 of the CLSI antibiotics recommended for slowly growing mycobacteria. Thus, 2 of the 5 NTM isolated in this study presented MDR profile, and were more drug resistant than the previously isolated strains of the same species. Multidrug-resistant NTM have been described in the literature but not originating from the hospital environment [9, 50]. Intraspecific variability, infrequent isolation and lack of reports on drug resistance profiles all contribute to the difficulty in defining standard treatment guidelines for rare opportunistic NTM such as *M. mucogenicum* [51]. Remarkably, all NTM isolates in this study were susceptible to the aminoglycosides tested, except for the 2 rapidly growing NTM isolates that were resistant to tobramycin.

*Corynebacterium* are in general resistant to antimicrobial agents recommended for Gram-positive infections, including penicillins, cephalosporins, macrolides, fluoroquinolones, aminoglycosides and tetracycline, but they remain susceptible to vancomycin and linezolid [52]. The 3 *Corynebacterium* isolates in this study showed an unusual resistance pattern, since they were susceptible to aminoglycosides and tetracyclines, but were also resistant to linezolid. Riegel et al. tested the susceptibility of 13 nosocomial *C. jeikeium* isolates against gentamicin, with half presenting a MIC< 0.5 μg/mL and the other half a MIC> 16 μg/mL [53]. Scarce literature with low number of isolates hinders overall interpretation of results, which may be worth exploring for further awareness of antibiotic resistance in these increasingly detected potentially opportunistic pathogens.

One important observation from our study was the fact that all *Corynebacterium* and *Gordonia* isolates were highly resistant to imipenem. Although we did not assess the genetic background underlying this phenotype, it is possible to speculate that if the resistance is related to the presence of carbapenemases, these slowly growing bacteria can represent an unknown resistance pool against this important antibiotic. We found no literature reporting the presence of carbapenemases in species of the *Corynebacterineae*, but genetic mobile elements with different resistance genes have already been described in some of these species [54, 55], highlighting their ability to transfer antibiotic resistance features.

Only 5 of the 25 tested antimicrobials were those used in Middlebrook 7H10-PANTA culture medium for isolation although our results showed MICs much higher than the concentrations used in the selective medium. Possibly, increasing the concentration of these antimicrobials, except azlocillin, could diminish the number of false positives for the selection of NTM from the hospital environment, as the presence of a higher number of colony forming units per plate observed in some samples could have affected the recovery rate (data not shown). This is worth testing to optimize present standard methodology for NTM isolation with PANTA-supplemented medium [56]. Surprisingly, while *Corynebacterium* and *Gordonia* isolates were highly resistant to the antifungal amphotericin B, the MICs for most NTM isolates were intermediate confirming that at higher concentrations amphotericin B seems to inhibit their growth.

## Conclusions

The current study, although limited in number of isolates, revealed the poor knowledge we still have on the identity of viable NTM species present in hospital settings, as well as on their antibiotic resistance profiles and resistance mechanisms, raising relevant questions about the potential threat these and other potential opportunistic pathogens may represent for example to immunocompromised inpatients. Their presence in dry surfaces with which healthcare providers, visitors and patients themselves contact frequently, accompanied by their apparent multidrug resistance profiles, should be further investigated to comprehensively understand this potentially latent menace and help prevent dissemination through implementation of better disinfection strategies and enforcement of enhanced policies.

## Materials and methods

### Sample collection from hospital settings

Samples were collected from different surfaces and equipment located at three different wards of a tertiary care hospital, as previously described [17]. Suspensions without pre-treatment were directly plated in solid Middlebrook 7H10-PANTA supplemented medium [Middlebrook 7H10 medium enriched with 10% OADC (oleic acid, albumin, dextrose, catalase) and supplemented with polymyxin B (40 U/mL), amphotericin B (4 μg/mL), nalidixic acid (16 μg/mL), trimethoprim (4 μg/mL) and azlocillin (4 μg/mL)] [17, 56]. Plates were incubated at 30 °C between 1 and 6 weeks and colony growth was evaluated on a weekly basis. Isolation, plating and purification of colonies was performed in Middlebrook 7H10-PANTA, followed by cryopreservation at − 80 °C in Middlebrook 7H9 broth with 15% glycerol.

### Identification of NTM and other actinobacterial isolates

Genomic DNA was extracted as previously described [57]. Amplification of the full-length 16S rRNA gene was performed by polymerase chain reaction (PCR) with universal primers 27F (5′-GAGTTTGATCCTGGCTCAG) and 1525R (5′–AGAAAGGAGGTGATCCAGCC) [58]. PCR reactions were carried out with Supreme NZYTaq DNA polymerase (NZYTech, Portugal) with 30 cycles of 1 min at 94 °C, 1 min at 55 °C, and 1 min at 72 °C. Products were purified using JET Quick PCR Purification Spin Kit (Genomed GmbH, Germany) according to manufacturer’s instructions and sequenced at GATC Biotech (Germany). 16S rRNA gene sequences were compared with sequences at the NCBI database using the BLAST tool (http://blast.ncbi.nlm.nih.gov/) and assignment to species level considered nucleotide sequence identities of ≥99%. For species identity validation, DNA from *Mycobacterium* isolates was used for PCR amplification of partial sequences of *rpoB* and *hsp65* genes with mycobacterial-specific primers GrpoB1 (5′-ATCGACCACTTCGGCAACCGCC), GrpoB2 (5′-GGTACGGCGTCTCGATGAASCCG), and Tb11 (5′-ACCACGATGGTGTGTCCAT), Tb12 (5′-CTTGTCGAACCGCATACCCT), respectively [59]. PCR reactions were carried out with KOD Hot-Start DNA polymerase (Novagen) according to manufacturer’s instructions and PCR products were purified and sequenced, as described above.

### Sequence analyses and phylogenetic trees

Phylogenetic analyses were performed after manually checking DNA quality using Sequence Scanner Software (Applied Biosystems). Sequence data was edited and assembled with BioEdit Sequence Alignment Editor. The 16S rRNA gene sequences of the isolates and type strains of the *Corynebacterineae* genera *Mycobacterium*, *Gordonia* and *Corynebacterium* were obtained from Genbank or ARB Silva database (https://www.arb-silva.de/) and aligned, each genus separately, with the Clustal X software package [60], visually examined and manually adjusted to allow maximal alignment. Jukes Cantor method was used to calculate evolutionary distances [61]. Phylogenetic dendrograms were constructed by the neighbor-joining method and evaluated by bootstrap analysis [62] of 500 resamplings of the data set, using MEGA6 software [63]. Three phylogenetic trees (Additional file 1: Figure S1, Additional file 2: Figure S2, Additional file 3: Figure S3, Additional file 4: Figure S4 and Additional file 5: Figure S5) were used to accurately determine the phylogenetic placement of the isolates for downstream selection of the Type strains to be used for the construction of the final tree (as described above) including the three genera belonging to *Corynebacterineae* (Fig. 1). The similarity values of the 16S rRNA gene sequences of the all isolates and the closest type strains were determined from the alignment used to construct the phylogenetic tree encompassing the three genera and are presented as Additional file 6: Table S1. The assignment to species level considered nucleotide sequence similarity value of ≥99% of the isolates towards the closest type strains. Amino acid sequences were deduced with the MEGA6 package from the 420- and 396-bp DNA sequences of mycobacterial partial *hsp65* and *rpoB* gene sequences, respectively. Protein sequences were aligned with sequences of type strains obtained from the NCBI database using the Clustal X. Protein phylogenetic trees were constructed using the neighbor-joining [64]. Topology of trees were generated from evolutionary distances computed using the Poisson correction method [65], included in Mega6 and evaluated by bootstrap analysis [62] of 500 resamplings of the data set. All positions with less than 95% site coverage were eliminated. Protein alignments were used to determine the nucleotide position in the DNA sequences alignment and sequences from mycobacterial genes 16S rRNA, *hsp65* and *rpoB* were concatenated and further used for phylogenetic analyses as described above.

To search for the clarithromycin resistance gene *erm* and for the tetracycline resistance gene *tet(V)*, chromosomal DNA from NTM isolates was used as template for draft genome sequencing at GATC Biotech (Konstanz, Germany) with 150 bp paired-end libraries on an Illumina HiSeq. Raw sequence reads were assembled de novo using SPAdes 3.11.1 [66] with specific parameters for 2 × 150 bp reads library de novo assembly, namely using BayesHammer module error correction and --careful option (our unpublished results).

### Deposition of nucleic acid sequences in public databases

Partial 16S rRNA (1347–1378 bp) genes are available from [17] under accession numbers KT347497 and KT347499 to KT347502. Partial *rpoB* (371–398 bp) and *hsp65* (395–441 bp) genes sequences were deposited in European Molecular Biology Laboratory (EMBL) and GenBank databases under the accession numbers: KT992215 to KT992224 for the partial *rpoB* and *hsp65* sequences, respectively, and from KT832812 to KT832816 for the *Corynebacterineae* isolates partial 16S rRNA sequences. The clarithromycin resistance gene *erm* detected in the *M. obuense* genome and the tetracycline resistance genes *tet(V)* identified in *M. obuense* and *M. mucogenicum* draft genomes were deposited in GenBank database under accession numbers MG770427, MG770425 and MG770428, respectively.

### Antimicrobial susceptibility testing and minimal inhibitory concentration (MIC)

Minimal inhibitory concentrations were determined after 5 days according to Clinical Laboratory Standards Institute (CLSI) recommendations for rapidly growing NTM and *Nocardia* [18, 67]. *Corynebacterium* isolates were incubated for 48 h according to CLSI recommendations [68]. The slowly growing *M. paragordonae* isolates 10AIII, 29AIII and 35AIII were incubated for 10 days. Clarithromycin susceptibility was determined after 14 days [69]. Classification of mycobacteria according to their growth rate is classically based on the time bacteria take to form colonies in solid media. Rapidly growing mycobacteria (RGM) are able to grow in under 7 days, whereas the ones that take more than 7 days are called slowly growing mycobacteria (SGM). Because phylogenetic studies of mycobacteria support this separation identification of SGM or RGM species was based on the phylogenetic tree constructed by Tortoli et al. [70] in addition to CLSI listing [18]. Briefly, a suspension of 0.5 McFarland density of each isolate was prepared in saline solution and diluted 1000-fold before testing in the next 30 min. A sterile 96-well microplate, previously prepared with Mueller Hinton (MH) medium supplemented with 0.5% OADC and containing decreasing concentrations of the tested antimicrobials, was inoculated with the diluted bacterial suspension and incubated for 5 days at 30 °C [18]. In addition to the antimicrobials considered for rapidly growing mycobacteria susceptibility testing in CLSI standards (cefoxitin, amikacin, imipenem, tobramycin, linezolid, doxycycline, clarithromycin and ciprofloxacin) also amphotericin B, azlocillin, nalidixic acid, trimethoprim, polymyxin B (these 5 used in Middlebrook 7H10-PANTA), rifampicin, chloramphenicol, tetracycline, penicillin, vancomycin and the aminoglycosides gentamicin, kanamycin, streptomycin, neomycin and spectinomycin were tested. Only antimicrobials considered in CLSI standards were interpreted for bacterial resistance levels [18, 68]. Diverse concentration ranges were used, with antimicrobials being diluted 8 times, in a 1:2 scaling (clarithromycin was diluted 12 times and penicillin was diluted 16 times). Stock solutions were prepared according CLSI guidelines [71, 72]. Appropriate controls were performed to ensure normal bacterial growth despite presence of diluted acetic acid, methanol or ethanol used to solubilize some antibiotics. All assays were performed in triplicate.

## Additional files

Additional file 1: **Figure S1.** Phylogenetic dendrogram constructed by comparing 16S rRNA gene sequences of isolates 10AIII, 22DIII, 24AIII, 29AIII and 35AIII with *Mycobacterium* type strain sequences obtained from GenBank databases. Sequences were aligned using MEGA6. The tree topology was obtained by using neighbor-joining algorithm with Jukes–Cantor correction. All positions with less than 95% site coverage were eliminated. Bootstrap values above 60%, for 500 replicates, are given at branch points. Bar, 1 inferred nucleotide substitution per 100 nt. (PPTX 108 kb)

Additional file 2: **Figure S2.** Phylogenetic dendrogram constructed by comparing 16S rRNA gene sequences of isolates 1AIII and 55AIII with *Gordonia* type strain sequences obtained from databases. Sequences were aligned using MEGA6. The tree topology was obtained by using neighbor-joining algorithm with Jukes–Cantor correction. All positions with less than 95% site coverage were eliminated. Bootstrap values above 60%, for 500 replicates, are given at branch points. Bar, 5 inferred nucleotide substitution per 1000 nt. (PPTX 75 kb)

Additional file 3: **Figure S3.** Phylogenetic dendrogram constructed by comparing 16S rRNA gene sequences of isolates 6FIII, 52AIII and 58FIII with *Corynebacterium* type strain sequences obtained from databases. Sequences were aligned using MEGA6. The tree topology was obtained by using neighbor-joining algorithm with Jukes–Cantor correction. All positions with less than 95% site coverage were eliminated. Bootstrap values above 60%, for 500 replicates, are given at branch points. Bar, 1 inferred nucleotide substitution per 100 nt. (PPTX 95 kb)

Additional file 4: **Figure S4.** Phylogenetic analysis of *rpoB* nucleotide sequences of mycobacterial isolates and 61 selected types strains of the genus *Mycobacterium*. The tree was created using the neighbor-joining algorithm and the evolutionary distances calculated by Jukes and Cantor method [61]. Bootstrap values above 60%, for 500 replicates, are given at branch points. Bar, 1 inferred nucleotide substitution per 100 nt. (PPTX 92 kb)

Additional file 5: **Figure S5.** Phylogenetic analysis of *hsp65* nucleotide sequences of mycobacterial isolates and the 55 selected type strains of the genus *Mycobacterium*. See Additional file 5: Figure S2 legend for further details. (PPTX 90 kb)

Additional file 6: **Table S1.** Pairwise similarity values (%) determined from the alignment used for the construction of the phylogenetic trees from: A. The concatenated nucleotide sequences of mycobacterial 16S rRNA, *hsp65* and *rpoB* of isolates and Type strains selected from the databases; B. 16S rRNA gene nucleotide sequences of isolates and Type strains of the genus Corynebacterium selected from the databases; C. 16S rRNA gene nucleotide sequences of isolates and Type strains of the genus *Gordonia* selected from the databases. (DOCX 48 kb)

## Abbreviations

BLAST: Basic Local Alignment Search Tool
CFU: Colony forming units
CLSI: Clinical Laboratory Standards Institute
EMBL: European Molecular Biology Laboratory
HAI: Hospital-acquired infections
MDR: Multidrug resistance
MIC: Minimal inhibitory concentration
NCBI: National Center for Biotechnology Information
NTM: Nontuberculous mycobacteria
OADC: Oleic acid, albumin, dextrose, catalase
PANTA: polymyxin B (40 U/mL), amphotericin B (4 μg/mL), nalidixic acid (16 μg/mL), trimethoprim (4 μg/mL) and azlocillin
PCR: Polymerase chain reaction
WHO: World Health Organization

## References

[1] WHO (2011). Report on the Burden of Endemic Health Care-Associated Infection Worldwide.

[2] Allegranzi B, Kilpatrick C, Storr J, Kelley E, Park BJ, Donaldson L, Global Infection P, Control N (2017). Global infection prevention and control priorities 2018-22: a call for action. Lancet Glob Health.

[3] ECDC (2017). Healthcare-associated infections acquired in intensive care units - annual epidemiological report 2016 [2014 data]. Annual Epidemiological Report on Communicable Diseases in Europe.

[4] Boucher HW, Talbot GH, Bradley JS, Edwards JE, Gilbert D, Rice LB, Scheld M, Spellberg B, Bartlett J (2009). Bad Bugs, No Drugs: No ESKAPE! An update from the Infectious Diseases Society of America. Clin Infect Dis.

[5] WHO (2017). Global priority list of antibiotic-resistant bacteria to guide research, discovery, and development of new antibiotics.

[6] Fedrizzi T, Meehan CJ, Grottola A, Giacobazzi E, Fregni Serpini G, Tagliazucchi S, Fabio A, Bettua C, Bertorelli R, De Sanctis V (2017). Genomic characterization of nontuberculous mycobacteria. Sci Rep.

[7] Falkinham JO (2016). Current epidemiologic trends of the nontuberculous mycobacteria (NTM). Curr Environ Health Rep.

[8] Griffith DE, Aksamit T, Brown-Elliott BA, Catanzaro A, Daley C, Gordin F, Holland SM, Horsburgh R, Huitt G, Iademarco MF (2007). An official ATS/IDSA statement: diagnosis, treatment, and prevention of nontuberculous mycobacterial diseases. Am J Respir Crit Care Med.

[9] Brown-Elliott BA, Nash KA, Wallace RJ (2012). Antimicrobial susceptibility testing, drug resistance mechanisms, and therapy of infections with nontuberculous mycobacteria. Clin Microbiol Rev.

[10] Tuttle MS, Mostow E, Mukherjee P, Hu FZ, Melton-Kreft R, Ehrlich GD, Dowd SE, Ghannoum MA (2011). Characterization of bacterial communities in venous insufficiency wounds by use of conventional culture and molecular diagnostic methods. J Clin Microbiol.

[11] Russotto V, Cortegiani A, Raineri SM, Giarratano A (2015). Bacterial contamination of inanimate surfaces and equipment in the intensive care unit. J Intensive Care.

[12] LPSN—list of prokaryotic names with standing in nomenclature. 1997. www.bacterio.net/mycobacterium.html. Accessed 10 Dec 2018. 10.1093/nar/gkt1111 PMC3965054 24243842

[13] Iida S, Taniguchi H, Kageyama A, Yazawa K, Chibana H, Murata S, Nomura F, Kroppenstedt RM, Mikami Y (2005). *Gordonia otitidis* sp. nov., isolated from a patient with external otitis. Int J Syst Evol Microbiol.

[14] Biswal I, Mohapatra S, Deb M, Dawar R, Gaind R (2014). *Corynebacterium striatum*: an emerging nosocomial pathogen in a case of laryngeal carcinoma. Indian J Med Microbiol.

[15] van der Werf M, Kodmon C, Katalinic-Jankovic V, Kummik T, Soini H, Richter E, Papaventsis D, Tortoli E, Perrin M, van Soolingen D (2014). Inventory study of non-tuberculous mycobacteria in the European Union. BMC Infect Dis.

[16] Nunes-Costa D, Alarico S, Dalcolmo MP, Correia-Neves M, Empadinhas N (2016). The looming tide of nontuberculous mycobacterial infections in Portugal and Brazil. Tuberculosis..

[17] Geadas Farias P, Gama F, Reis D, Alarico S, Empadinhas N, Martins JC, de Almeida AF, Morais PV (2017). Hospital microbial surface colonization revealed during monitoring of *Klebsiella* spp., *Pseudomonas aeruginosa*, and non-tuberculous mycobacteria. Antonie Van Leeuwenhoek.

[18] CLSI (2011). Susceptibility testing of Mycobacteria, Nocardia, and other aerobic Actinomycetes; approved standard. In., vol. document M24-A, 2nd edn.

[19] Cummings KL, Anderson DJ, Kaye KS (2010). Hand hygiene noncompliance and the cost of hospital-acquired methicillin-resistant *Staphylococcus aureus* infection. Infect Control Hosp Epidemiol.

[20] Lai CC, Wang CY, Liu CY, Tan CK, Lin SH, Liao CH, Chou CH, Huang YT, Lin HI, Hsueh PR (2010). Infections caused by Gordonia species at a medical Centre in Taiwan, 1997 to 2008. Clin Microbiol Infect.

[21] Adekambi T (2009). *Mycobacterium mucogenicum* group infections: a review. Clin Microbiol Infect.

[22] Belmares J, Detterline S, Pak JB, Parada JP (2007). Corynebacterium endocarditis species-specific risk factors and outcomes. BMC Infect Dis.

[23] Weber DJ, Rutala WA, Miller MB, Huslage K, Sickbert-Bennett E (2010). Role of hospital surfaces in the transmission of emerging health care-associated pathogens: norovirus, Clostridium difficile, and Acinetobacter species. Am J Infect Control.

[24] Bryant JM, Grogono DM, Rodriguez-Rincon D, Everall I, Brown KP, Moreno P, Verma D, Hill E, Drijkoningen J, Gilligan P (2016). Emergence and spread of a human-transmissible multidrug-resistant nontuberculous mycobacterium. Science..

[25] Walker TM, Crook DW, Peto TEA, Conlon CP (2016). Whole-genome sequencing identifies nosocomial transmission of extra-pulmonary *M. tuberculosis*. QJM..

[26] Crago B, Ferrato C, Drews SJ, Louie T, Ceri H, Turner RJ, Roles A, Louie M (2014). Surveillance and molecular characterization of non-tuberculous mycobacteria in a hospital water distribution system over a three-year period. J Hosp Infect.

[27] Genc GE, Richter E, Erturan Z (2013). Isolation of nontuberculous mycobacteria from hospital waters in Turkey. APMIS..

[28] Sartori FG, Leandro LF, Montanari LB, de Souza MGM, Pires RH, Sato DN, Leite CQF, de Andrade PK, Martins CHG (2013). Isolation and identification of environmental mycobacteria in the waters of a hemodialysis center. Curr Microbiol.

[29] Fernandez-Rendon E, Cerna-Cortes JF, Ramirez-Medina MA, Helguera-Repetto AC, Rivera-Gutierrez S, Estrada-Garcia T, Gonzalez-y-Merchand JA (2012). *Mycobacterium mucogenicum* and other non-tuberculous mycobacteria in potable water of a trauma hospital: a potential source for human infection. J Hosp Infect.

[30] Souza MG, Sato DN, Leite CQF, Leite SR, Sartori FG, Prince K, Casmeiro LA, Martins CH (2010). Occurrence of pathogenic environmental mycobacteria on surfaces in health institutions. Res Rep Trop Med.

[31] Shin JH, Lee EJ, Lee HR, Ryu SM, Kim HR, Chang CL, Kim YJ, Lee JN (2007). Prevalence of non-tuberculous mycobacteria in a hospital environment. J Hosp Infect.

[32] Falkinham JO, Pruden A, Edwards M (2015). Opportunistic premise plumbing pathogens: increasingly important pathogens in drinking water. Pathogens..

[33] Archuleta R, Mullens P, Primm TP (2002). The relationship of temperature to desiccation and starvation tolerance of the Mycobacterium avium complex. Arch Microbiol.

[34] Kim BJ, Hong SH, Kook YH, Kim BJ (2014). *Mycobacterium paragordonae* sp. nov., a slowly growing, scotochromogenic species closely related to *Mycobacterium gordonae*. Int J Syst Evol Microbiol.

[35] Azadi D, Shojaei H, Pourchangiz M, Dibaj R, Davarpanah M, Naser AD (2016). Species diversity and molecular characterization of nontuberculous mycobacteria in hospital water system of a developing country. Iran Microb Pathog.

[36] Schreiber PW, Kuster SP, Hasse B, Bayard C, Rüegg C, Kohler P, Keller PM, Bloemberg GV, Maisano F, Bettex D (2016). Reemergence of mycobacterium chimaera in heater–cooler units despite intensified cleaning and disinfection protocol. Emerg Infect Dis.

[37] Ruas RR, Abreu I, Nuak J, Ramos A, Carvalho T, Ribeiro M, Guimaraes JT, Sarmento A (2017). Nontuberculous mycobacteria in a tertiary Hospital in Portugal: a clinical review. Int J Mycobacteriol.

[38] Koskimäki JJ, Hankala E, Suorsa M, Nylund S, Pirttilä AM (2010). Mycobacteria are hidden endophytes in the shoots of rock plant [Pogonatherum paniceum (Lam.) Hack.] (Poaceae). Environ Microbiol Rep.

[39] Laukkanen H, Soini H, Kontunen-Soppela S, Hohtola A, Viljanen M (2000). A mycobacterium isolated from tissue cultures of mature Pinus sylvestris interferes with growth of scots pine seedlings. Tree Physiol.

[40] Tsukamura M, Mizuno S (1971). *Mycobacterium obuense*, a rapidly growing scotochromogenic mycobacterium capable of forming a black product from *p*-aminosalicylate and salicylate. J Gen Microbiol.

[41] Ford ES, Horne DJ, Shah JA, Wallis CK, Fang FC, Hawn TR (2017). Species-specific risk factors, treatment decisions, and clinical outcomes for laboratory isolates of less common nontuberculous mycobacteria in Washington state. Ann Am Thorac Soc.

[42] Greninger AL, Cunningham G, Hsu ED, Yu JM, Chiu CY, Miller S (2015). Draft genome sequence of *Mycobacterium obuense* strain UC1, isolated from patient sputum. Genome Announc.

[43] Buijtels PCAM, Iseman MD, Parkinson S, de Graaff CS, Verbrugh HA, Petit PLC, van Soolingen D (2010). Misdiagnosis of tuberculosis and the clinical relevance of non—tuberculous mycobacteria in Zambia. Asian Pac J Trop Med.

[44] Martin-Casabona N, Bahrmand AR, Bennedsen J, Thomsen VO, Curcio M, Fauville-Dufaux M, Feldman K, Havelkova M, Katila ML, Koksalan K (2004). Non-tuberculous mycobacteria: patterns of isolation. A multi-country retrospective survey. Int J Tuberc Lung Dis.

[45] Kim J, Seong M-W, Kim E-C, Han SK, Yim J-J (2015). Frequency and clinical implications of the isolation of rare nontuberculous mycobacteria. BMC Infect Dis.

[46] Brown-Elliott BA, Philley JV. Rapidly Growing Mycobacteria. Microbiol Spectr. 2017;5(1). http://www.asmscience.org/content/journal/microbiolspec/10.1128/microbiolspec.TNMI7-0027-2016. 10.1128/microbiolspec.tnmi7-0027-2016 PMC11687460 28084211

[47] Li G, Pang H, Guo Q, Huang M, Tan Y, Li C, Wei J, Xia Y, Jiang Y, Zhao X (2017). Antimicrobial susceptibility and MIC distribution of 41 drugs against clinical isolates from China and reference strains of nontuberculous mycobacteria. Int J Antimicrob Agents.

[48] van Ingen J, van der Laan T, Dekhuijzen R, Boeree M, van Soolingen D (2010). In vitro drug susceptibility of 2275 clinical non-tuberculous Mycobacterium isolates of 49 species in the Netherlands. Int J Antimicrob Agents.

[49] Soetaert K, Rens C, Wang X-M, De Bruyn J, Lanéelle M-A, Laval F, Lemassu A, Daffé M, Bifani P, Fontaine V (2015). Increased vancomycin susceptibility in mycobacteria: a new approach to identify synergistic activity against multidrug-resistant mycobacteria. Antimicrob Agents Chemother.

[50] Candido PH, Nunes LS, Marques EA, Folescu TW, Coelho FS, de Moura VC, da Silva MG, Gomes KM, Lourenco MC, Aguiar FS (2014). Multidrug-resistant nontuberculous mycobacteria isolated from cystic fibrosis patients. J Clin Microbiol.

[51] Brown-Elliott BA, Wallace RJ. Clinical and taxonomic status of pathogenic nonpigmented or late-pigmenting rapidly growing mycobacteria. Clin Microbiol Rev. 2002;15(4):716-46. 10.1128/CMR.15.4.716-746.2002 PMC126856 12364376

[52] Humphries RM, Hindler JA, Jorgensen JH, Pfaller MA, Carroll KC, Funke G, Landry ML, Richter SS, Warnock DW (2015). Susceptibility test methods: fastidious bacteria. Manual of clinical microbiology.

[53] Riegel P, de Briel D, Prévost G, Jehl F, Monteil H (1994). Genomic diversity among *Corynebacterium jeikeium* strains and comparison with biochemical characteristics and antimicrobial susceptibilities. J Clin Microbiol.

[54] Tauch A, Krieft S, Pühler A, Kalinowski J (1999). The tetAB genes of the *Corynebacterium striatum* R-plasmid pTP10 encode an ABC transporter and confer tetracycline, oxytetracycline and oxacillin resistance in Corynebacterium glutamicum. FEMS Microbiol Lett.

[55] Tauch A, Krieft S, Kalinowski J, Puhler A (2000). The 51,409-bp R-plasmid pTP10 from the multiresistant clinical isolate *Corynebacterium striatum* M82B is composed of DNA segments initially identified in soil bacteria and in plant, animal, and human pathogens. Mol Gen Genet.

[56] Radomski N, Cambau E, Moulin L, Haenn S, Moilleron R, Lucas FS (2010). Comparison of culture methods for isolation of nontuberculous mycobacteria from surface waters. Appl Environ Microbiol.

[57] Alarico S, Costa M, Sousa MS, Maranha A, Lourenco EC, Faria TQ, Ventura MR, Empadinhas N (2014). *Mycobacterium hassiacum* recovers from nitrogen starvation with up-regulation of a novel glucosylglycerate hydrolase and depletion of the accumulated glucosylglycerate. Sci Rep.

[58] Rainey FA, Ward-Rainey N, Kroppenstedt RM, Stackebrandt E (1996). The genus *Nocardiopsis* represents a phylogenetically coherent taxon and a distinct actinomycete lineage: proposal of Nocardiopsaceae fam. nov. Int J Syst Bacteriol.

[59] Devulder G, de Montclos MP, Flandrois JP (2005). A multigene approach to phylogenetic analysis using the genus *Mycobacterium* as a model. Int J Syst Evol Microbiol.

[60] Larkin MA, Blackshields G, Brown NP, Chenna R, McGettigan PA, McWilliam H, Valentin F, Wallace IM, Wilm A, Lopez R (2007). Clustal W and Clustal X version 2.0. Bioinformatics..

[61] Jukes TH, Cantor CR, Munro HN (1969). Evolution of protein molecules. Mammalian protein metabolism.

[62] Felsenstein J (1985). Confidence limits on phylogenies: an approach using the bootstrap. Evolution.

[63] Tamura K, Stecher G, Peterson D, Filipski A, Kumar S (2013). MEGA6: molecular evolutionary genetics analysis version 6.0. Mol Biol Evol.

[64] Saitou N, Nei M (1987). The neighbor-joining method: a new method for reconstructing phylogenetic trees. Mol Biol Evol.

[65] Zuckerkandl E, Pauling L, Bryson V, Vogel HJ (1965). Evolutionary divergence and convergence in proteins. Evolving genes and proteins.

[66] Bankevich A, Nurk S, Antipov D, Gurevich AA, Dvorkin M, Kulikov AS, Lesin VM, Nikolenko SI, Pham S, Prjibelski AD (2012). SPAdes: a new genome assembly algorithm and its applications to single-cell sequencing. J Comput Biol.

[67] Woods GL, Lin S-YG, Desmond EP, Jorgensen JH, Pfaller MA, Carroll KC, Funke G, Landry ML, Richter SS, Warnock DW (2015). Susceptibility test methods: mycobacteria, nocardia, and other actinomycetes. Manual of clinical microbiology.

[68] CLSI (2015). Methods for antimicrobial dilution and disk susceptibility testing of infrequently isolated or fastidious bacteria. In., 3rd edn.

[69] Brown-Elliott BA, Vasireddy S, Vasireddy R, Iakhiaeva E, Howard ST, Nash K, Parodi N, Strong A, Gee M, Smith T (2015). Utility of sequencing the *erm (41)* gene in isolates of *Mycobacterium abscessus subsp. abscessus* with low and intermediate clarithromycin MICs. J Clin Microbiol.

[70] Tortoli E, Fedrizzi T, Meehan CJ, Trovato A, Grottola A, Giacobazzi E, Serpini GF, Tagliazucchi S, Fabio A, Bettua C (2017). The new phylogeny of the genus Mycobacterium: the old and the news. Infect Genet Evol.

[71] Barry A, Bryskier A, Traczewski M, Brown S (2004). Preparation of stock solutions of macrolide and ketolide compounds for antimicrobial susceptibility tests. Clin Microbiol Infect.

[72] CLSI (2017). Performance standards for antimicrobial susceptibility testing.

