# Supplementary figures and images for: Studies of antimicrobial resistance in rare mycobacteria from a nosocomial environment
Source: BMC Microbiol. 2019 Mar 19;19:62. doi: 10.1186/s12866-019-1428-4 (PMC6425705; doi:10.1186/s12866-019-1428-4)

## Slide 1
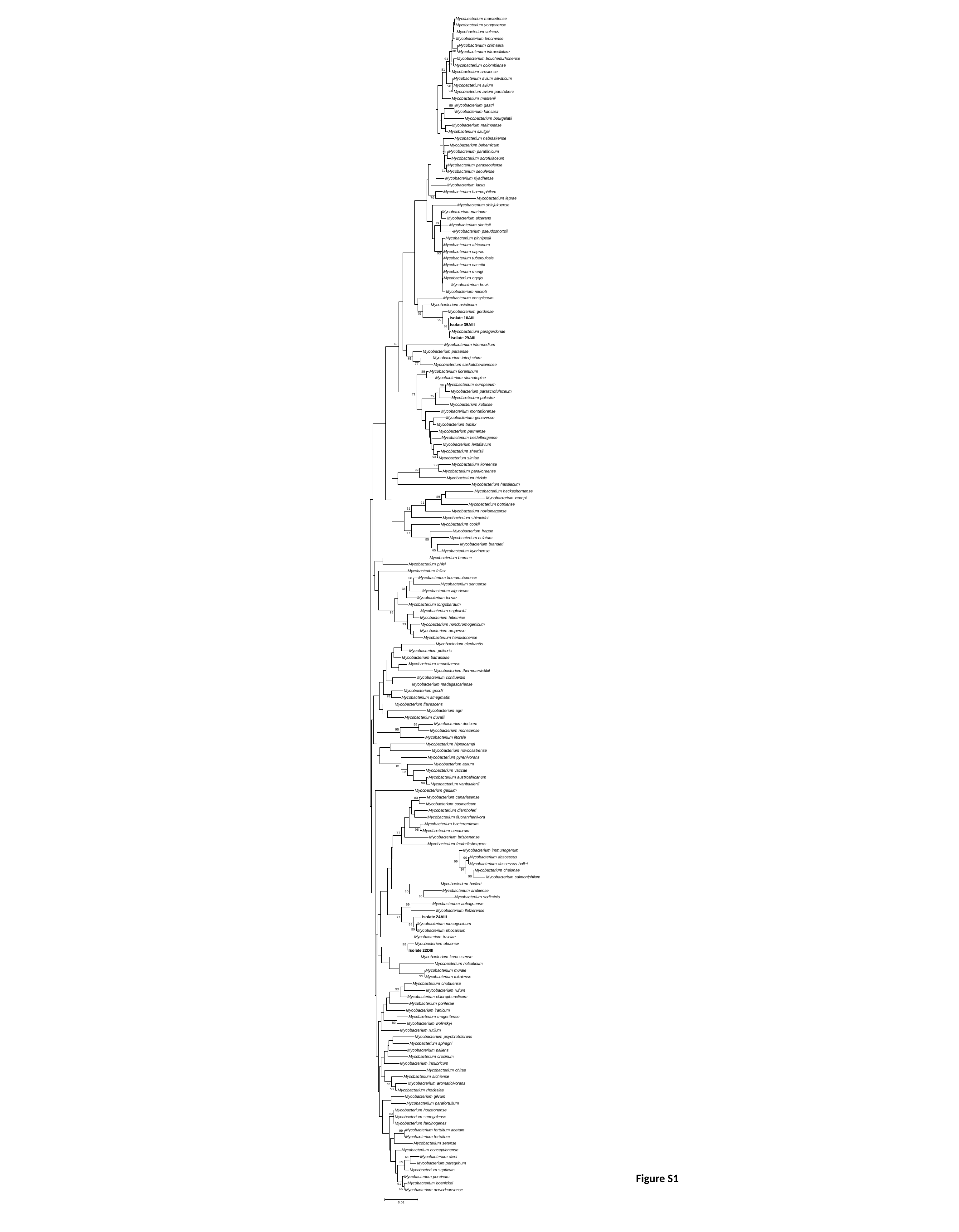

Figure S1

Supplement: Supplementary file 1 — Figure S1. Phylogenetic dendrogram constructed by comparing 16S rRNA gene sequences of isolates 10AIII, 22DIII, 24AIII, 29AIII and 35AIII with Mycobacterium type strain sequences obtained from GenBank databases. Sequences were aligned using MEGA6. The tree topology was obtained by using neighbor-joining algorithm with Jukes–Cantor correction. All positions with less than 95% site coverage were eliminated. Bootstrap values above 60%, for 500 replicates, are given at branch points. Bar, 1 inferred nucleotide substitution per 100 nt. (PPTX 108 kb) [file 12866_2019_1428_MOESM1_ESM.pptx]

## Slide 1
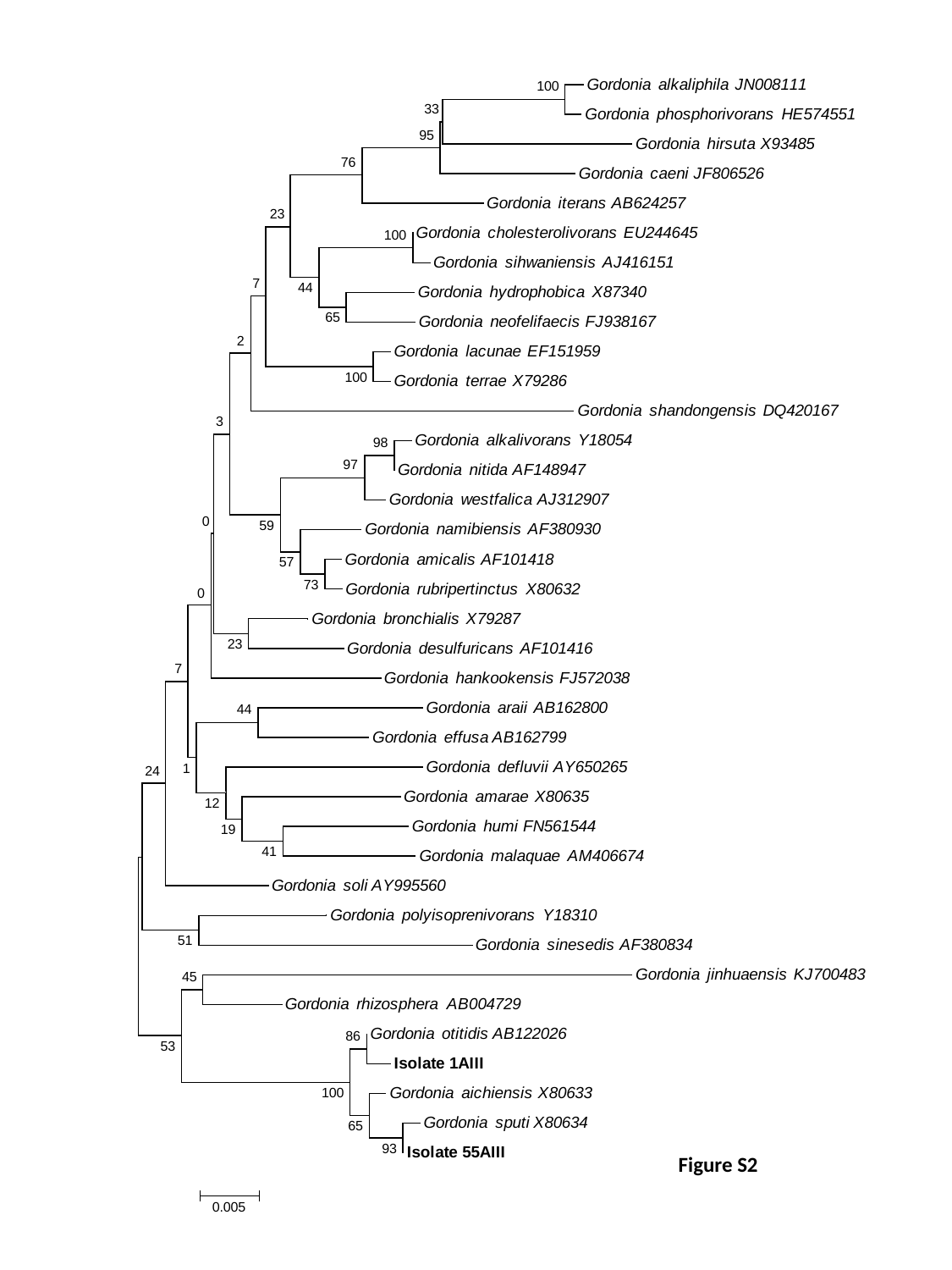

Figure S2

Supplement: Supplementary file 2 — Figure S2. Phylogenetic dendrogram constructed by comparing 16S rRNA gene sequences of isolates 1AIII and 55AIII with Gordonia type strain sequences obtained from databases. Sequences were aligned using MEGA6. The tree topology was obtained by using neighbor-joining algorithm with Jukes–Cantor correction. All positions with less than 95% site coverage were eliminated. Bootstrap values above 60%, for 500 replicates, are given at branch points. Bar, 5 inferred nucleotide substitution per 1000 nt. (PPTX 75 kb) [file 12866_2019_1428_MOESM2_ESM.pptx]

## Slide 1
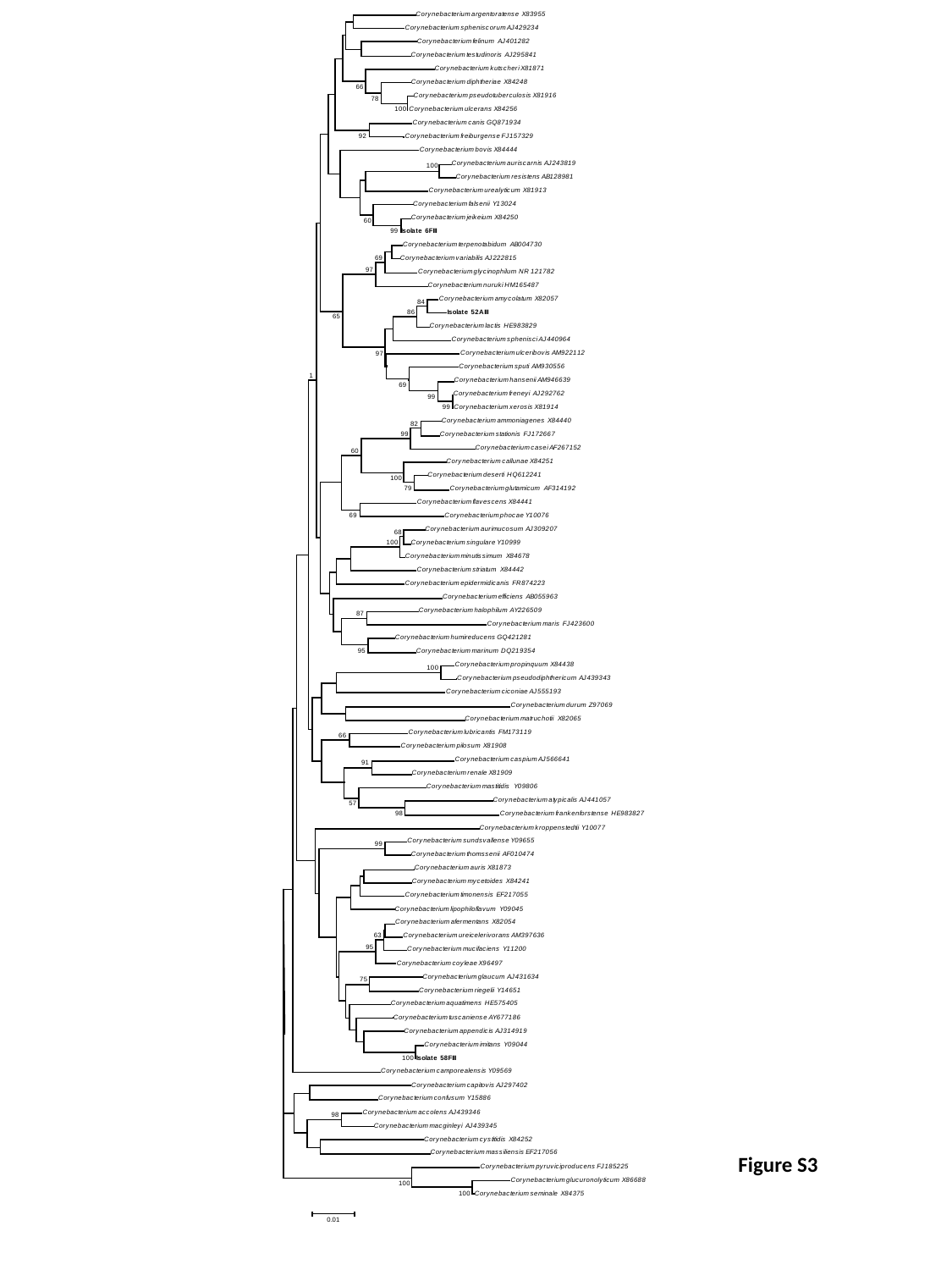

Figure S3

Supplement: Supplementary file 3 — Figure S3. Phylogenetic dendrogram constructed by comparing 16S rRNA gene sequences of isolates 6FIII, 52AIII and 58FIII with Corynebacterium type strain sequences obtained from databases. Sequences were aligned using MEGA6. The tree topology was obtained by using neighbor-joining algorithm with Jukes–Cantor correction. All positions with less than 95% site coverage were eliminated. Bootstrap values above 60%, for 500 replicates, are given at branch points. Bar, 1 inferred nucleotide substitution per 100 nt. (PPTX 95 kb) [file 12866_2019_1428_MOESM3_ESM.pptx]

## Slide 1
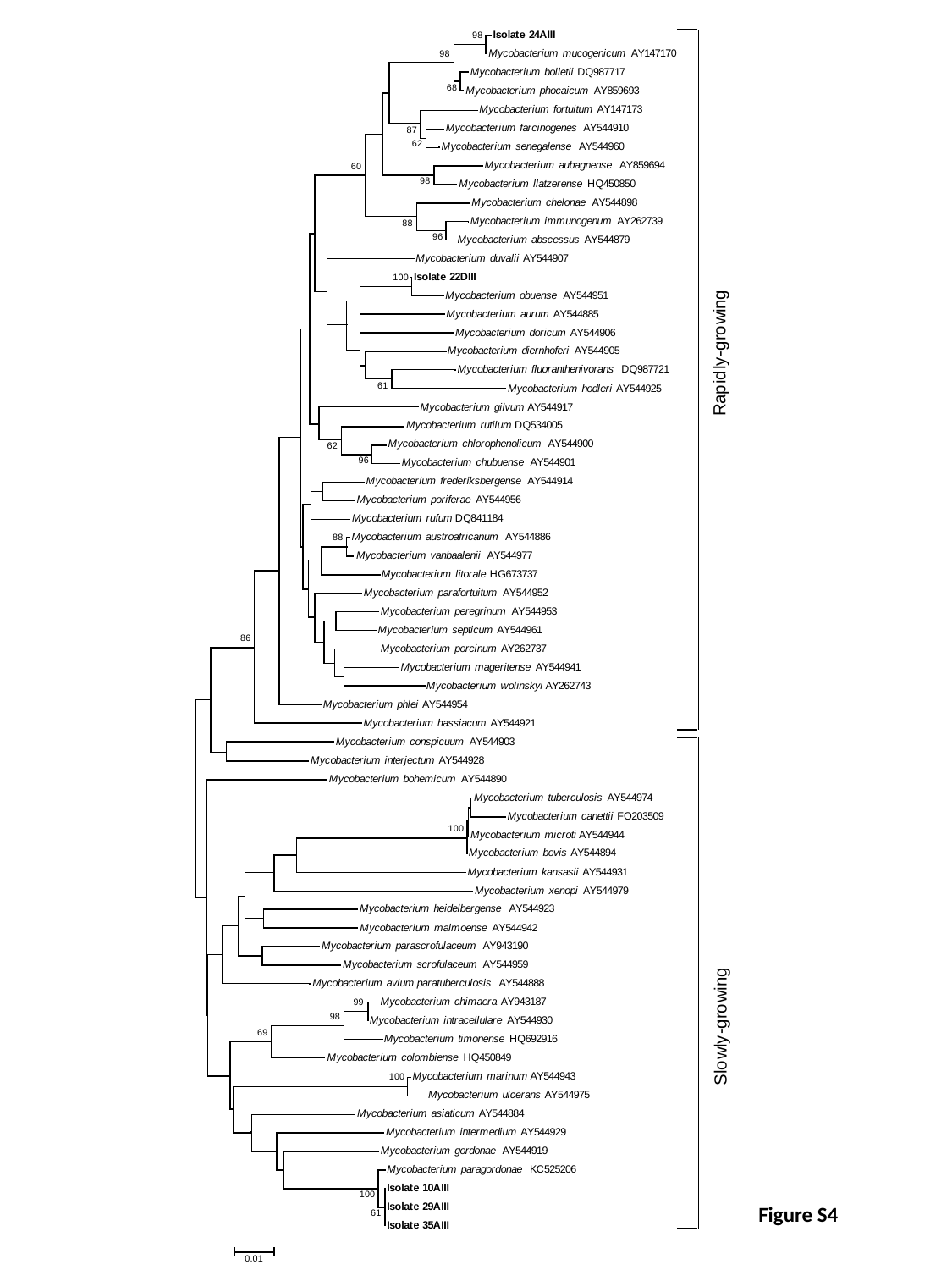

Figure S4

Supplement: Supplementary file 4 — Figure S4. Phylogenetic analysis of rpoB nucleotide sequences of mycobacterial isolates and 61 selected types strains of the genus Mycobacterium. The tree was created using the neighbor-joining algorithm and the evolutionary distances calculated by Jukes and Cantor method [61]. Bootstrap values above 60%, for 500 replicates, are given at branch points. Bar, 1 inferred nucleotide substitution per 100 nt. (PPTX 92 kb) [file 12866_2019_1428_MOESM4_ESM.pptx]

## Slide 1
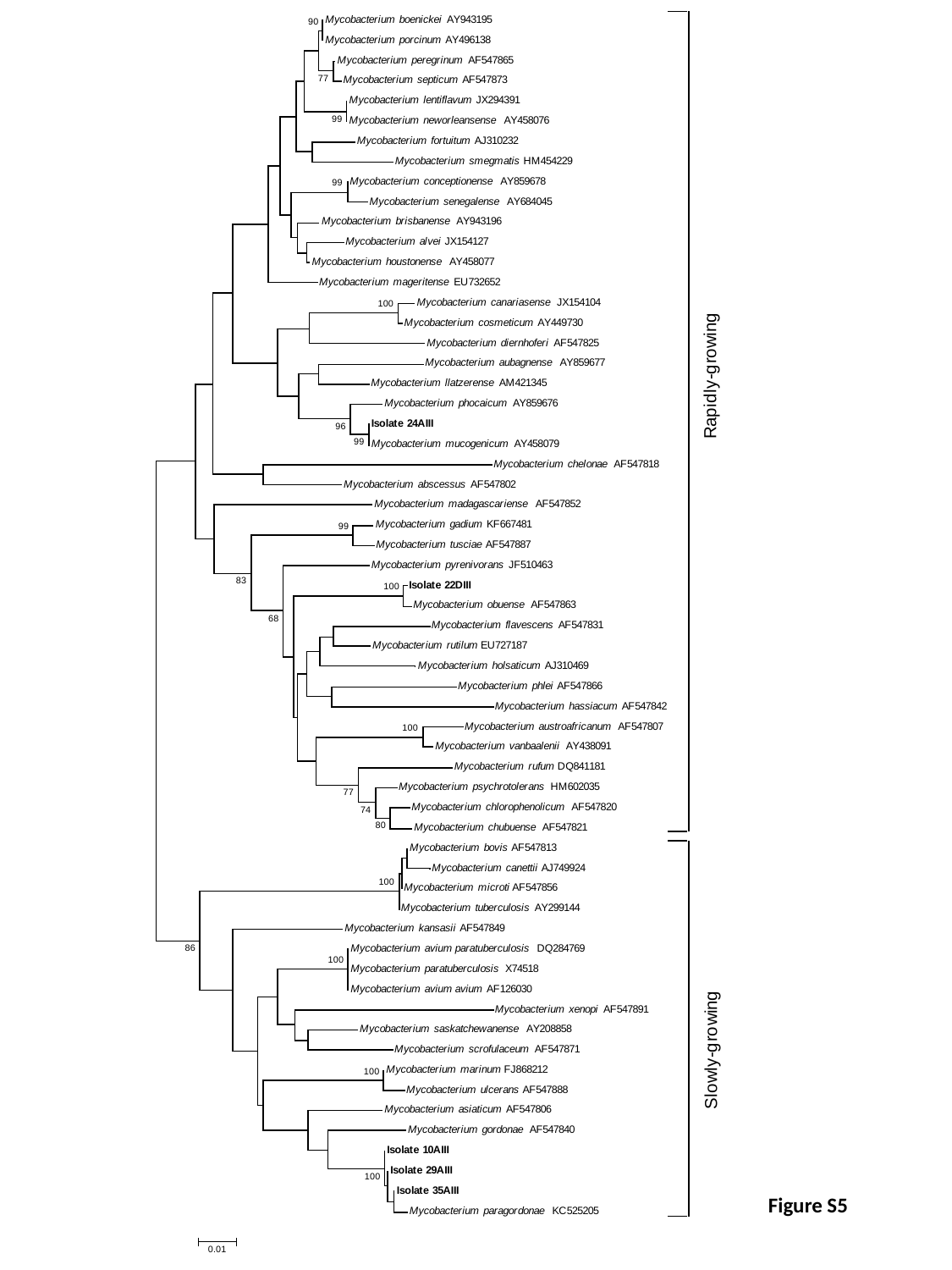

Figure S5

Supplement: Supplementary file 5 — Figure S5. Phylogenetic analysis of hsp65 nucleotide sequences of mycobacterial isolates and the 55 selected type strains of the genus Mycobacterium. See Additional file 5: Figure S2 legend for further details. (PPTX 90 kb) [file 12866_2019_1428_MOESM5_ESM.pptx]
