# Supplementary material for: Studies of antimicrobial resistance in rare mycobacteria from a nosocomial environment
Source: BMC Microbiol. 2019 Mar 19;19:62. doi: 10.1186/s12866-019-1428-4 (PMC6425705; doi:10.1186/s12866-019-1428-4)
Supplement: Supplementary file 6 — Table S1. Pairwise similarity values (%) determined from the alignment used for the construction of the phylogenetic trees from: A. The concatenated nucleotide sequences of mycobacterial 16S rRNA, hsp65 and rpoB of isolates and Type strains selected from the databases; B. 16S rRNA gene nucleotide sequences of isolates and Type strains of the genus Corynebacterium selected from the databases; C. 16S rRNA gene nucleotide sequences of isolates and Type strains of the genus Gordonia selected from the databases. (DOCX 48 kb) [file 12866_2019_1428_MOESM6_ESM.docx]

**Table S1** - Pairwise similarity values (%) determined from the alignment used for the construction of the phylogenetic trees from: **A**. The concatenated nucleotide sequences of mycobacterial 16S rRNA, *hsp65* and *rpoB* of isolates and Type strains selected from the databases; **B.** 16S rRNA gene nucleotide sequences of isolates and Type strains of the genus *Corynebacterium* selected from the databases; **C.** 16S rRNA gene nucleotide sequences of isolates and Type strains of the genus *Gordonia* selected from the databases.

| **A** |  |  |  |  |  |
| --- | --- | --- | --- | --- | --- |
|  | **Isolate 10AIII** | **Isolate 22DIII** | **Isolate 24AIII** | **Isolate 29AIII** | **Isolate 35AIII** |
| *Mycobacterium mucogenicum* | 94.2 | 96.0 | 99.8 | 94.2 | 94.2 |
| *Mycobacterium obuense* | 93.5 | 99.7 | 95.5 | 93.5 | 93.5 |
| *Mycobacterium paragordonae* | 100 | 93.7 | 94.1 | 100 | 100 |
|  |  |  |  |  |  |
| **B** |  |  |  |  |  |
|  | **Isolate 55AIII** | **Isolate 1AIII** |  |  |  |
| *Gordonia otitidis* | 99.6 | 100 |  |  |  |
| *Gordonia sputi* | 99.9 | 99.4 |  |  |  |
|  |  |  |  |  |  |
| **C** |  |  |  |  |  |
|  | **Isolate 6FIII** | **Isolate 52AIII** | **Isolate 58FIII** |  |  |
| *Corynebacterium amycolatum* | 95.0 | 99.2 | 94.6 |  |  |
| *Corynebacterium imitans* | 93.6 | 94.1 | 99.9 |  |  |
| *Corynebacterium jeikeium* | 100 | 94.6 | 93.4 |  |  |
|  |  |  |  |  |  |
